# A comment on: ‘Absorbed radiation doses in the thyroid as estimated by UNSCEAR and subsequent risk of childhood thyroid cancer following the Great East Japan’

**DOI:** 10.1093/jrr/rraa145

**Published:** 2021-02-10

**Authors:** Hidehiko Yamamoto, Keiji Hayashi, Hagen Scherb

**Affiliations:** Osaka Red Cross Hospital attached facility of physically handicapped children, 5-30 Fudegasaki-cho, Tennouji-ku Osaka-Shi 543-8555 Osaka, Japan; Hayashi Children’s Clinic, 4-6-11-1F Nagata, Joto-ku Osaka-Shi 536-0022 Osaka, Japan

## Dear Editor

We were very interested in the article by Ohira *et al*. [1]. Whereas Tsuda *et al*. [2], Yamamoto *et al*. [3], Kato [4] and Toki *et al*. [5] found a significant association between the occurrence of thyroid cancer and radiation following the Fukushima nuclear accidents, Ohira *et al*. claim no association between thyroid doses and thyroid cancer risk.

Ohira *et al*. [1] stratified the Fukushima prefecture into four regions defined by the quartiles of the absorbed thyroid dose distribution and assumed that the dose should have been avoided in the evacuation areas. The question arises of whether the evaluation of the thyroid dose including the evacuated municipalities can show a significant correlation. To this end, we considered the municipality-specific counts of thyroid cancers and the person-years in the Fukushima Health Management Survey (FHMS) as published in tables 1 and 2 of Yamamoto *et al*. [3]. Table 1 supplements this information with the total absorbed thyroid dose to 10-year-old children as estimated by UNSCEAR in the Attachments C-16 and C-18 of its 2013 Report [6]. These internal doses are compiled in the last column of Table 1, whereby the missing dose values in Attachment C-16 for the partly or completely evacuated prefectures were imputed by the dose values in Attachment C-18 taking the proportion of evacuees in the individual municipalities into account by linear interpolation.

**Table 1:** FHMS basic data of the combined first and second screening rounds [3]: municipality, person-years, thyroid cancers, detection rate and UNSCEAR (2013) total thyroid absorbed dose of 10-year-old children (mGy) in the first year after Fukushima derived from the UNSCEAR 2013 Report Attachments C-16 and C018 [6]

Location no.	Municipality	Person-years	Thyroid cancers	Detection rate per 100 000	Total thyroid dose for 10-year-old children (mGy)^a^
1	Kawamata Machi	5790	2	34.54	29.04
2	Namie Machi	8304	4	48.17	83.75
3	Iitate Mura	2502	0	0.00	55.92
4	Minamisoma Shi	29 333	6	20.45	35.32
5	Date Shi	30 411	9	29.59	22.61
6	Tamura Shi	17 133	5	29.18	19.42
7	Hirono Machi	2359	0	0.00	41.19
8	Naraha Machi	3401	0	0.00	85.26
9	Tomioka Machi	6812	1	14.68	121.31
10	Kawauchi Mura	755	1	132.45	41.32
11	Okuma Machi	5933	3	50.56	112.68
12	Futaba Machi	2475	0	0.00	28.72
13	Katsurao Mura	521	0	0.00	67.17
14	Fukushima Shi	146 213	22	15.05	28.73
15	Nihonmatsu Shi	29 623	6	20.25	27.41
16	Motomiya Shi	17 788	6	33.73	21.00
17	Otama Mura	4777	2	41.87	23.96
18	Koriyama Shi	192 018	43	22.39	22.82
19	Koori Machi	6298	1	15.88	24.72
20	Kunimi Machi	4808	0	0.00	19.61
21	Ten-ei Mura	3009	0	0.00	20.47
22	Shirakawa Shi	36 846	7	19.00	18.81
23	Nishigo Mura	12 499	2	16.00	19.69
24	Izumizaki Mura	3954	1	25.29	18.08
25	Miharu Machi	9695	1	10.31	19.87
26	Iwaki Shi	195 353	31	15.87	31.16
27	Sukagawa Shi	48 513	5	10.31	18.82
28	Soma Shi	20 546	1	4.87	17.47
29	Kagamiishi Machi	8262	1	12.10	17.85
30	Shinchi Machi	4515	0	0.00	17.26
31	Nakajima Mura	3524	1	28.38	16.39
32	Yabuki Machi	11 354	1	8.81	16.86
33	Ishikawa Machi	9559	1	10.46	15.80
34	Yamatsuri Machi	3500	0	0.00	15.59
35	Asakawa Machi	4840	0	0.00	16.36
36	Hirata Mura	3929	1	25.45	16.30
37	Tanagura Machi	10 042	2	19.92	17.30
38	Hanawa Machi	5526	1	18.10	16.23
39	Samegawa Mura	2317	0	0.00	16.39
40	Ono Machi	6237	0	0.00	16.54
41	Tamakawa Mura	4513	0	0.00	15.99
42	Furudono Machi	3677	0	0.00	16.37
43	Hinoemata Mura	300	0	0.00	15.32
44	Minamiaizu Machi	8288	0	0.00	15.45
45	Kaneyama Machi	612	0	0.00	15.41
46	Showa Mura	447	0	0.00	15.80
47	Mishima Machi	574	0	0.00	15.97
48	Shimogo Machi	3047	1	32.82	15.40
49	Kitakata Shi	26 455	3	11.34	18.44
50	Nishiaizu Machi	2968	0	0.00	15.58
51	Tadami Machi	2220	1	45.05	16.03
52	Inawashiro Machi	8435	1	11.86	16.53
53	Bandai Machi	1893	0	0.00	16.61
54	Kitashiobara Mura	1752	0	0.00	19.46
55	Aizumisato Machi	11 713	1	8.54	16.10
56	Aizubange Machi	9570	1	10.45	19.90
57	Yanaizu Machi	1755	0	0.00	15.91
58	Aizuwakamatsu Shi	67 951	8	11.77	16.64
59	Yugawa Mura	2342	1	42.70	18.46
**Total or mean**		**1 079 786**	**184**	**17.04**	**26.96**

^a^Derived from the UNSCEAR 2013 Report Attachments C-16 and C-18 [6]

**Table 2:** Dose ranges, range-specific mean values of dose, thyroid cancer cases, person-years and detection rates (DR^r^ raw and DR^a^ adjusted) derived from the study of Lubin *et al*. [10] and detection rate (DR) from the study of Yamamoto *et al*. [3]

Dose range (mGy)	Lubin *et al*. (2017)					Yamamoto *et al*. (2019)			
	Mean (mGy)	Cases	Person-years	DR^r^	DR^a^	Mean (mGy)	Cases	Person-years	DR
0	0	142	1 865 957	7.6	7.6	–	–	–	–
1–4	2	24	367 606	6.5	8.1	–	–	–	–
4–20	9	30	587 614	5.1	9.2	17	47	386 111	12.2
20–40	25	13	345 748	3.8	6.6	26	128	663 088	19.3
40–60	49	54	315 014	17.1	15.3	46	1	5616	17.8
60–80	68	31	256 456	12.1	10.7	67	0	521	0.0
80–100	88	32	242 247	13.2	13.5	85	4	11 705	34.2
100–120	107	20	136 943	14.6	19.1	113	3	5933	50.6
120–140	126	21	149 525	14.0	20.0	121	1	6812	14.7
140–160	146	13	73 824	17.6	28.6	–	–	–	–
160–190	177	14	113 582	12.3	18.3	–	–	–	–
**Total**		394	4 454 516			–	184	1 079 786	–

**Fig. 1.**
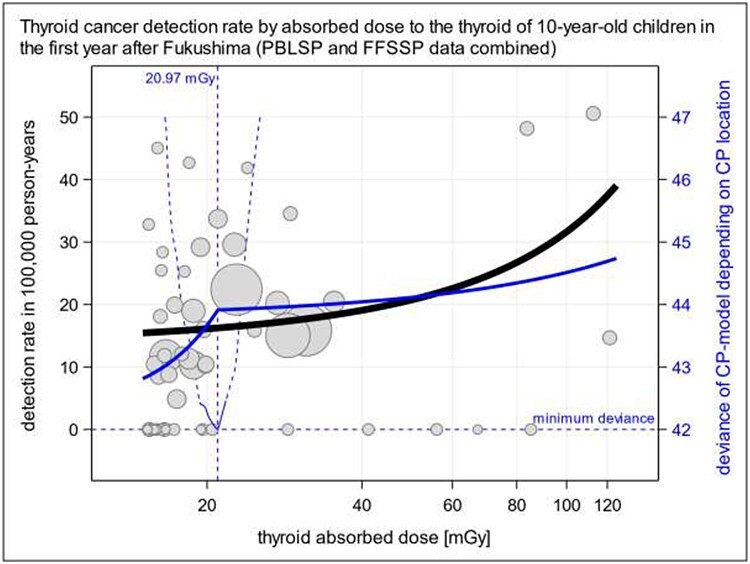
Association between thyroid cancer detection rate and thyroid absorbed dose (mGy) in 59 municipalities of Fukushima after the nuclear accidents (see Table 1). Thick solid black line: overall Poisson regression of the detection rate on the absorbed dose. Dashed blue lines: estimation of optimum change point of segmented regression [7]. Solid blue line, segmented Poisson regression of the detection rate on the absorbed dose allowing for an optimum change of slope at 21 mGy; outlying data point Kawauchi Mura not shown; circle area is proportional to expected thyroid cancer cases; PBLSP, Primary Base Line Screening Program, FFSSP First Full-Scale Screening Program.

Yamamoto *et al*. [3] found a considerably elevated detection rate per dose-rate of thyroid cancer below 2 μSv h^–1^ compared with the detection rate ratio from unrestricted data. We built on this finding by performing a segmented regression analysis [7] to determine an optimum dose (mGy) beyond which the slope of the detection rate by dose changes significantly. The dashed light blue elements in Fig. 1 present the corresponding change point analysis based on the deviance criterion [8]. The optimum thyroid absorbed dose of this change point is 21 mGy, 95% confidence interval (CI) 17–24. The detection rate ratio (DRR) below 21 mGy is 1.154 per mGy, 95% CI 1.044–1.277, *P* value 0.0053, and the residual DRR above 21 mGy is 1.003. The odds ratio and the *P* value for the interaction (change of slope) are 0.869, 95% CI 0.783–0.965, and 0.0083, respectively. This means that the overall effect is driven by the strong effect below 21 mGy. The solid blue line in Fig. 1 depicts this change point model. The solid black line in Fig. 1 indicates the overall association between the thyroid cancer occurrence and the thyroid absorbed dose in all 59 municipalities of Fukushima after the nuclear accidents. The DRR and the *P* value for this overall trend are 1.008, 95% CI 1.000–1.017, and 0.0445, respectively. The first- and second-order models are possible alternatives, which cannot be distinguished with certainty based on the data at hand. The presence of significant non-linearity does not mean that a simple linear overall model is inappropriate. If the simple linear model is not significant, this is not evidence of no effect [9].

**Fig. 2.**
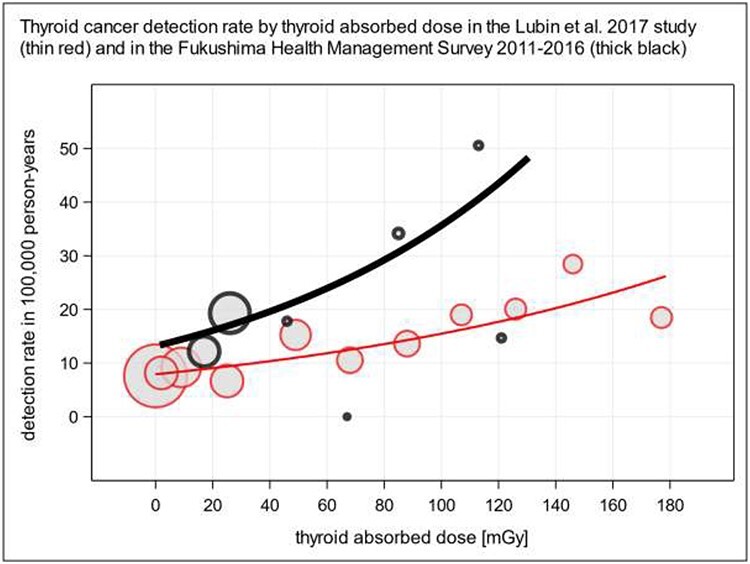
Adjusted thyroid cancer detection rate by thyroid absorbed dose derived from the study of Lubin *et al*. [10] (see Table 2): thin red line and circles. Detection rate from the study of Yamamoto *et al*. [3]: thick black line and circles. Circle areas proportional to person-years for dose categories. The detection rate ratios (DRRs) per mGy and their 95% confidence intervals are 1.0067, 1.0046-1.0088, *P* value < 0.0001 for the study of Lubin *et al*. [7], and 1.0100, 1.0006-1.0196, *P* value 0.0379 for the FHMS [3].

The raw detection rate (DR^r^ = cases/person-years) and of the adjusted detection rate (DR^a^ = RR^a^ × cases^0^/person-years^0^), where superscript ‘0’ means the counts of cases (*n* = 142) and person-years (*n* = 1 865 957) at zero dose can be determined using table 1 in Lubin *et al*. [10]. These data are compiled in Table 2 and depicted in Fig. 2 comparing the detection rates of Lubin *et al*. and Yamamoto *et al*. DRRs per mGy, 95% CIs and *P* values of the trends in Fig. 2 are 1.0067, 1.0046–1.0088 and < 0.0001 for Lubin *et al*. [10], and 1.0100, 1.0006–1.0196 and 0.0379 for Yamamoto *et al*. [3]. Therefore, the meta-analysis of Lubin *et al*. and the FHMS yield consistent relative risks of the order of magnitude of 1% per 1 mGy thyroid absorbed dose in 10-year-old children. Yamamoto *et al*. found an association between radiation and thyroid cancer within 5 years after the Fukushima nuclear accidents. In contrast, Lubin *et al*. state ‘Although data were limited, fitted RRs in the restricted data appeared compatible with a minimum latency of 5 to 10 years’. Veiga *et al*. support this finding [11]. However, these estimates of the minimum latency are based on few observations and cannot entirely exclude the possibility of earlier disease onset in (unnoticed) highly exposed or particularly sensitive children, see also paragraph ‘2.2 Induction and latent period, point prevalence, incidence proportion and incidence rate, and detection rate’ in Yamamoto *et al*. [3].

In summary, our findings contradict the conclusion of Ohira *et al*. stating ‘No dose-dependent pattern emerged from the geographical distribution of absorbed doses by municipality, as estimated by UNSCEAR, and the detection of thyroid cancer among participants within 4–6 years after the accident’ [1]. We conjecture that the negative finding by Ohira *et al*. [1] may partly be due to a too coarse exposure stratification, the neglect of the evacuation areas and the disregard of the non-linearity of the association between radiation dose and thyroid cancer in the FHMS.

## CONFLICT OF INTEREST

The authors declare that they have no known conflicts of interest.
